# Diagnostic utility of clinical characteristics, laboratory tests, and serum ferritin in diagnosis of adult-onset Still disease

**DOI:** 10.1097/MD.0000000000030152

**Published:** 2022-08-26

**Authors:** Iftach Sagy, Alona Finkel-Oron, Eviatar Naamany, Leonid Barski, Mahmoud Abu-Shakra, Yair Molad, Shachaf Shiber

**Affiliations:** a Rheumatic Diseases Unit, Soroka University Medical Center, Beer Sheva, Israel; b Internal Medicine Division, Soroka University Medical Center, Beer Sheva, Israel; c Faculty of Health Sciences, Ben-Gurion University of the Negev, Beer Sheva, Israel; d Institute of Rheumatology, Rabin Medical Center, Beilinsone Hospital, Petach Tikva, Israel; e Internal Medicine Division, Rabin Medical Center, Beilinsone Hospital, Petach Tikva, Israel; f Sackler Faculty of Medicine, Tel Aviv University, Tel Aviv, Israel.

**Keywords:** Adult onset Still disease, diagnostic utility, ferritin

## Abstract

The diagnosis of adult-onset Still disease (AOSD) is challenging with ambiguous clinical presentation and no specific serological markers. We aim to evaluate the diagnostic utility of clinical, laboratory and serum ferritin features in established AOSD patients. We included all patients >18 years who were admitted to 2 tertiary medical centers (2003–2019) with serum ferritin above 1000 ng/mL. AOSD patients and non-AOSD controls were matched in 1:4 ratio for age and sex. The primary outcomes were sensitivity, specificity, positive/negative likelihood ratio and area under the curve (AUC) using clinical and laboratory characteristics based on the Yamaguchi classification criteria, in addition to serum ferritin. We identified 2658 patients with serum ferritin above 1000 ng/m, of whom 36 diagnosed with AOSD and 144 non-AOSD matched controls. Presence of arthralgia/arthritis showed the highest sensitivity (0.74), specificity (0.93), positive likelihood ratio (10.69), negative likelihood ratio (0.27) and AUC (0.83, 95% confidence interval 0.74–0.92) to the diagnosis of AOSD. On the other hand, serum ferritin showed variation and poorer results, depends on the chosen ferritin cutoff. Joint involvement showed the best diagnostic utility to establish the diagnosis of AOSD. Although clinicians use often elevated ferritin levels as an anchor to AOSD, the final diagnosis should be based on thorough clinical evaluation.

## 1. Introduction

Adult onset Still disease (AOSD) is a rare systemic autoimmune disorder, with a prevalence of 1 to 10 per million.^[1,2]^ AOSD is characterized by spiking fever, typical evanescent rash, joint involvement, sore throat, and elevated acute phase reactants.^[3]^ Some of these clinical features are related to high levels of serum interleukin 6.^[4]^ In addition, extremely elevated serum ferritin levels are also observed among AOSD patients.^[5]^ The sharp elevation of serum ferritin level is explained by interleukin-1β-induced mRNA ferritin synthesis in hepatocytes and interleukin-18-induced ferritin release from splenic macrophages.^[6,7]^

Six sets of classification criteria have been proposed to establish for the classification of AOSD, yet the Yamaguchi criteria considered the most commonly used in clinical practice.^[8,9]^ The Yamaguchi classification criteria composed of clinical features (eg, intermittent fever > 39°C for >1 week, arthralgia, typical rash, sore throat) and laboratory features (white blood cells > 10,000, elevated liver enzymes and negative results of serum antinuclear antibody or rheumatoid factor). A combination of 5 positive criteria (with at least 2 major criteria) is considered to have the highest sensitivity in patients with a definite diagnosis of AOSD.^[10]^ Although high levels of serum ferritin is an important diagnostic marker of AOSD, it is not included in the Yamaguchi criteria.^[5,11]^ Moreover, previous studies compared clinical and laboratory features between AOSD and non-AOSD cohorts, yet the contribution of each feature to establish the diagnosis has not been evaluated.^[11–13]^ Hence, the aim of the current study is to identify the exact role of clinical features, laboratory tests, and serum ferritin in establishing the diagnosis of AOSD.

## 2. Methods

### 2.1. Study population

Soroka University Medical Center (SUMC) is a tertiary medical center in the southern part of Israel (the Negev), and Rabin Medical Center (RMC), is a tertiary medical center in the center of Israel, both university-academic centers belong to the Clalit Health Services. The study included patients of age ≥18 years who were admitted to one of the 2 mentioned medical centers between 2003 and 2019 with at least 1 serum ferritin result above 1000 ng/mL (5-folds the upper normal limit). Patients were stratified into AOSD and non-AOSD groups based on the Yamaguchi classification criteria for AOSD.^[8]^ The diagnosis of AOSD was confirmed by a rheumatologist’s review of the electronic hospital chart and patients’ follow-up visit in the Outpatient Rheumatology clinic with no change of the discharge diagnosis.

### 2.2. Data extraction

The study was approved by SUMC and RMC institutional ethic committees. Since 2003, all health care records are electronic and available in a data warehouse for research purposes. We used the patients’ electronic charts as well as the Clalit Health Services comprehensive computerized data warehouse to extract demographic, medical and laboratory data. We retrieved laboratory data (eg, complete blood count, serum ferritin at admission, c-reactive protein, antinuclear antibodies) at baseline. Reason of admission and clinical characteristics relevant to AOSD diagnosis, were extracted from the electronic medical records of the index hospitalization, by 2 physicians. In addition, we extracted data of in-hospital mortality, intensive care unit admission and follow-up time (from index-hospitalization to time of death or the end of 2019). We continued data extraction up to 1 year from index hospitalization to capture 1-year mortality, maximal serum ferritin levels after discharge and final diagnosis to the non-AOSD group.

### 2.3. Statistical Analysis

Data are expressed as mean ± standard deviation (SD), median ± interquartile range (IQR) or number and percentage. We compared the baseline characteristics of patients stratified to AOSD and non-AOSD groups. We used the Chi-squared, the Mann-Whitney and t-tests to compare dichotomous, parametric, and continuous variables, respectively. Patients of the AOSD and non-AOSD groups were matched in a 1:4 ratio (caliper 0.001, greedy matching) adjusted for age and gender. The primary outcomes of this study were the sensitivity, specificity, positive likelihood ratio, and negative likelihood ratio values of the clinical features and laboratory tests based on the Yamaguchi classification criteria. In addition, we used several serum ferritin cutoffs based on previous studies who investigated the association of elevated ferritin levels and AOSD diagnosis.^[5,11,14]^ The performance characteristics of clinical and laboratory variables were analyzed using ROC curves. Data analysis was performed using SPSS version 25.0.

## 3. Results

A total of 2658 patients were admitted to SUMC and RMC during 2003 to 2019 with serum ferritin levels above 1000 ng/mL. We identified 36 patients who fulfilled Yamaguchi classification criteria for AOSD^[8]^ and matched 144 non-AOSD controls. Table 1 presents baseline characteristics of matched patients according to AOSD and non-AOSD groups. The AOSD patients had significantly lower rate of chronic kidney disease (0.0% vs 12.5%, *P* = .03) and malignancy (0.0% vs 19.6%, *P* = .002) at baseline. In addition, the AOSD patients presented more often with fever, arthralgia, rash, and sore throat (p<0.001 for all). Moreover, the AOSD group showed significantly lower risk for in-hospital mortality (0.0% vs 11.8%, *P* = .03) as well as for 1-year mortality (8.3% vs 25.0%, *P* = .04).

**Table 1:** General characteristics of adult-onset Still disease patients and matched cohort.

Variable	Matched control (n = 144)	AOSD (n = 36)	*P* value
Age (mean ± SD)	40.6 (18.5)	39.7 (19.4)	.78
Females (n, %)	98 (68.1)	25 (69.4)	1.00
Jewish (n, %)	90 (62.5)	27 (75.0)	.17
Ischemic heart disease (n, %)	9 (6.3)	1 (2.9)	.43
Chronic pulmonary obstructive disease (n, %)	2 (1.4)	1 (2.9)	.54
Hypertension (n, %)	33 (22.9)	5 (14.3)	.35
Heart failure (n, %)	15 (10.4)	2 (5.7)	.39
Diabetes (n, %)	24 (16.7)	6 (17.1)	1.00
Chronic kidney disease (n, %)	18 (12.5)	0 (0.0)	.03
Cerebrovascular accident (n, %)	7 (4.9)	1 (2.9)	.60
Malignancy (n, %)	28 (19.6)	0 (0.0)	.002
Smoker (n, %)	18 (12.5)	6 (17.1)	.47
Charlson index score (median, i.q range)	1.0 (0.0–4.0)	1.0 (0.0–2.0)	.10
Reason of admission (n, %)			
Fever	53 (36.8)	25 (71.4)	<.001
Rash	3 (2.1)	6 (7.1)	
Abdominal symptoms	29 (20.1)	0 (0.0)	
Other	59 (41.0)	4 (11.4)	
Additional symptoms (n, %)*			
Arthralgia or arthritis	10 (6.9)	26 (74.3)	<.001
Rash	10 (6.9)	22 (62.9)	<.001
Sore throat	15 (10.4)	20 (57.1)	<.001
Enlarge lymph nodes	25 (25.0)	15 (51.7)	.01
Hepatosplenomegaly	25 (23.6)	9 (33.3)	.32
Pleural effusion	22 (23.9)	9 (31.0)	.47
Pericardial effusion	6 (6.8)	3 (10.7)	.44
Neurological symptoms	10 (7.1)	2 (5.7)	.77
Final diagnosis			
Lymphoproliferative disease	13 (7.2)		
Solid tumor	8 (4.4)		
Infection	73 (40.6)		
Other	50 (27.8)		
Length of hospitalization, d (median, i.q range)	8.5 (4.1–16.7)	11.9 (6.5–16.5)	.27
ICU admission	30 (20.8)	4 (11.1)	.23
In-hospital mortality	17 (11.8)	0 (0.0)	.03
One-y mortality	36 (25.0)	3 (8.3)	.04
Follow-up time, y (median, i.q range)	3.3 (0.6–6.2)	4.8 (2.2–9.0)	.01

* Due to missing values data may not be added to 100%.

AOSD = adult-onset Still disease.

The laboratory characteristics are presented in Table 2. The AOSD group showed higher levels of baseline hemoglobin (11.3 ± 1.9 vs 9.7 ± 2.6 g/dL, *P* = .001), fibrinogen (712.2 ± 315.6 vs 531.9 ± 224.9 mg/mL, *P* = .01) and higher maximal ferritin level during index hospitalization (8496.0 vs 1757.0 ng/mL, *P* < .001).

**Table 2:** Laboratory characteristics of adult-onset Still disease patients and matched cohort.

Variable	Matched control (n = 144)	AOSD (n = 36)	*P* value
White blood cells, 10^9^ cells/L (mean ± SD)	11.1 (18.9)	12.9 (6.6)	.57
Platelets, 10^9^ cells/L (mean ± SD)	238.6 (185.3)	262.1 (180.4)	.50
Hemoglobin, g/dL (mean ± SD)	9.7 (2.6)	11.3 (1.9)	.001
C-reactive protein, mg/dL (mean ± SD)	13.0 (11.7)	16.8 (14.6)	.26
ESR, mm/h (median, i.q range)	44.0 (20.0–117.0)	96.0 (76.5–110.0)	.06
ALT, IU/L (mean ± SD)	127.2 (354.6)	53.3 (57.2)	.22
AST, IU/L (mean ± SD)	133.0 (362.2)	81.2 (98.5)	.40
Fibrinogen, mg/dL (mean ± SD)	531.9 (224.9)	712.2 (315.6)	.01
RF positive (n, %)*	6 (10.2)	1 (3.1)	.22
ANA positive (n, %)*	10 (15.9)	4 (12.1)	.62
Ferritin maximum during index hospitalization, ng/mL (median, i.q range)	1757.0 (1260.5–2659.0)	8496.0 (2121.0–14155.0)	<.001
Ferritin maximum during the following year, ng/mL (median, i.q range)	1646.0 (1256.0–2364.0)	1044.0 (122.5–4482.5)	.20

ALT = alanine aminotransferase, ANA =antinuclear antibody, AST = aspartate aminotransferase, ESR = erythrocyte sedimentation rate, RF = rheumatoid factor.

The diagnostic utility of clinical and laboratory characteristics for AOSD is presented in Table 3. Among the clinical features, arthralgia and/or arthritis had the highest sensitivity (74%) specificity (93%), positive likelihood ratio (10.69), negative likelihood ratio (0.27), and consequently highest AUC (0.83, 95% confidence interval 0.74–0.92). Among the laboratory features, high serum ferritin levels showed the best performance. The serum ferritin level above 2500 ng/mL had the highest sensitivity (74%) and negative likelihood ratio (0.35). The serum ferritin level above 5000 ng/mL had the highest AUC (0.77, 95% confidence interval 0.67–0.87). The serum ferritin level above 10,000 ng/mL had the highest specificity (97%) and positive likelihood ratio (16.11).

**Table 3:** Diagnostic utility of clinical and laboratory test for adult-onset Stills disease.

	Sensitivity	Specificity	Positive likelihood ratio	Negative likelihood ratio	AUC (95% CI)
Clinical features					
Fever > 39°C	0.71	0.63	1.94	0.45	0.67 (0.57–0.77)
Arthralgia or arthritis	0.74	0.93	10.69	0.27	0.83 (0.74–0.92)
Typical rash	0.62	0.93	9.05	0.4	0.78 (0.67–0.88)
Sore throat	0.57	0.89	5.48	0.47	0.73 (0.62–0.83)
Enlarge lymph nodes	0.51	0.75	2.06	0.64	0.63 (0.51–0.75)
Hepatosplenomegaly	0.33	0.76	1.41	0.87	0.54 (0.42–0.67)
Laboratory features					
White blood cells > 10 × 10^9^ cells/L	0.6	0.63	1.66	0.62	0.62 (0.51–0.72)
Elevated liver function	0.44	0.4	0.75	1.35	0.57 (0.46–0.67)
Negative ANA/RF	0.14	0.78	0.68	1.08	0.53 (0.41–0.65)
Ferritin > 2500 ng/mL	0.74	0.73	2.75	0.35	0.73 (0.64–0.83)
Ferritin > 5000 ng/mL	0.62	0.92	8.05	0.4	0.77 (0.67–0.87)
Ferritin > 10,000 ng/mL	0.45	0.97	16.11	0.55	0.71 (0.61–0.82)

## 4. Discussion

The principle finding of our study is that AOSD diagnosis is associated with a wide range of clinical and laboratory variability. To the best of our knowledge, this study is the first to compare AOSD patients to non-AOSD age- and sex-matched controls. Furthermore, we found in a matched analysis that arthritis/arthralgia and elevated serum ferritin levels have the highest diagnostic utility to diagnose AOSD based on the Yamaguchi classification criteria.^[8]^ However, while joint involvement showed the highest diagnostic utility in every parameter (sensitivity, specificity, positive/negative likelihood ratio, and AUC), the diagnostic utility of the serum ferritin level showed considerable variation and depended on the ferritin cutoff level.

AOSD is associated with elevated serum ferritin in 70% of cases.^[15]^ The increase in serum ferritin is attributed to an increase in proinflammatory cytokines production, specifically interleukin (IL)-1β and IL-18, and subsequent liver injury as well as macrophage activation.^[16,17]^ The combination of a glycosylated ferritin level of ≤ 20% with the serum ferritin level above the upper limit of normal range yielded a sensitivity of 70.5% and specificity of 83.2% for the diagnosis of AOSD, whereas the combination of a glycosylated ferritin level ≤ 20% with serum ferritin 5 times normal produced a sensitivity of 43.2% and specificity of 92.9%.^[14]^ A decrease in soluble IL-2 receptor after treatment initiation was associated with both decrease in serum ferritin levels and with higher chance to achieve low AOSD activity.^[18]^ Hence, our finding that higher levels of serum ferritin were observed in AOSD (compared to non-AOSD) only at index hospitalization (but not after 1 year of treatment), is consistent with the literature. One the other hand, several studies found that even high levels of serum ferritin levels (≥1000 ng/mL similar to the cutoff used in this analysis) have a poor positive predictive value for the diagnosis of AOSD, regardless of the threshold that have been used.^[14,19]^ Perhaps, from this reason, the Yamaguchi criteria does not include serum ferritin as a diagnostic criterion.

The diagnosis of AOSD is clinical. Unlike other rheumatic diseases, there is no specific laboratory marker to establish the diagnosis of AOSD, and the overall incline of acute phase reactants reflects AOSD-derived cytokines release and activated inflammatory state.^[20]^ From this point of view, the sharp rise in serum ferritin levels is part of the inflammatory state that couples with AOSD. Many clinicians consider the presence of high serum ferritin in the setting of fever of unknown origin as an important anchor to establish the diagnosis of AOSD.^[21]^ For instance, it has been proposed the serum ferritin is lower among patients with infectious disease-associated fever of unknown origin than in noninfectious inflammatory diseases.^[22]^ However, the diagnostic value of serum ferritin levels in AOSD was found to be relatively low. Fautrel et al reported that high serum ferritin levels produced only 40.8% sensitivity and 80.0% specificity to diagnose AOSD. The usefulness of serum ferritin level in establishing the diagnosis of AOSD is emphasized by the fact that other conditions such as hematological malignancies, iron overload, liver disease, acute infections, and other inflammatory conditions are associated with high levels of ferritin.^[23,24]^

Arthralgia and arthritis, on the other hand, are found in the majority of AOSD patients. Pouchot et al reported that among 62 AOSD patients there were 94% with arthritis and 100% with arthralgia.^[25]^ Other AOSD cohorts reported nearly 100% of arthralgia as well.^[15,26]^ Although we did not distinguish arthralgia from arthritis, 74.3% of our AOSD patients reported these symptoms, compared to only 6.9% in the non-AOSD group. The diagnostic utility of clinical joint involvement was found to surpass any other clinical or laboratory features, including high serum ferritin levels in our cohort. Thus, they clearly stress the importance relaying on clinical symptoms when establishing AOSD diagnosis.

This study has several limitations, which warrant further research. First, this study is of an observational nature and cannot establish causality between our results and AOSD diagnosis. Second, since we matched control only for age and sex, there may be sampling bias in our cohort. Third, due to lack of availability in our institutes, we did not use glycosylated ferritin, which has been previously reported to be a useful marker for the diagnosis of AOSD. Last, to our knowledge, this is the first study that analyzed sensitivity, specificity, likelihood ratio, and AUC of diagnostic tests in AOSD and matched controls. Hence, our results must be interpreted with cautious and validated in the future.

Notwithstanding these limitations, our study clearly delineates the importance of clinical symptoms, and more specifically joint involvement, in establishing the diagnosis of AOSD. High ferritin levels considered by many as an important marker of AOSD. Yet, our results emphasize that clinicians must be aware to other clinical features when they approach to a patient with suspected AOSD.

## References

[1] Wakai K Ohta A Tamakoshi A. Estimated prevalence and incidence of adult Still’s disease: findings by a nationwide epidemiological survey in Japan. J Epidemiol. 1997;7:221–5. 9465547 10.2188/jea.7.221

[2] Epidemiology of adult Still’s disease: estimate of the incidence by a retrospective study in West France. Annals of the Rheumatic Diseases [Internet]. Available at: https://ard.bmj.com/content/54/7/587.short [accessed date April 26, 2021]. 10.1136/ard.54.7.587 PMC1009940 7668903

[3] Larson EB. Adult Still’s disease—recognition of a clinical syndrome and recent experience. West J Med. 1985;142:665–71. 4013282 PMC1306135

[4] Hoshino T. Elevated serum interleukin 6, interferon-gamma, and tumor necrosis factor-alpha levels in patients with adult Still’s disease. J Rheumatol. 1998;25:396–8. 9489848

[5] Novak S Anic F Luke-Vrbanić TS. Extremely high serum ferritin levels as a main diagnostic tool of adult-onset Still’s disease. Rheumatol Int. 2012;32:1091–4. 21359498 10.1007/s00296-011-1834-x

[6] Rogers JT Bridges KR Durmowicz GP. Translational control during the acute phase response. Ferritin synthesis in response to interleukin-1. J Biol Chem. 1990;265:14572–8. 1696948

[7] Campbell CH Solgonick RM Linder MC. Translational regulation of ferritin synthesis in rat spleen: effects of iron and inflammation. Biochem Biophys Res Commun. 1989;160:453–9. 2470368 10.1016/0006-291x(89)92454-6

[8] Yamaguchi M Ohta A Tsunematsu T. Preliminary criteria for classification of adult Still’s disease. J Rheumatol. 1992;19:424–30. 1578458

[9] Kadavath S Efthimiou P. Adult-onset Still’s disease—pathogenesis, clinical manifestations, and new treatment options. 10. 10.3109/07853890.2014.971052 25613167

[10] Comparative Study of 6 Types of Criteria in Adult Still’s Disease. Abstract Europe PMC [Internet]. Available at: https://europepmc.org/article/med/8832990 [accessed date April 27, 2021].

[11] Lian F Wang Y Yang X. Clinical features and hyperferritinemia diagnostic cutoff points for AOSD based on ROC curve: a Chinese experience. Rheumatol Int. 2012;32:189–92. 20798946 10.1007/s00296-010-1601-4

[12] Comparison of clinical features of childhood and adult onset Still’s Disease. Abstract. Europe PMC [Internet]. Available at: https://europepmc.org/article/med/1767345 [accessed date April 27, 2021].

[13] Pay S Türkçapar N Kalyoncu M; Ankara Rheumatology Study Group. A multicenter study of patients with adult-onset Still’s disease compared with systemic juvenile idiopathic arthritis. Clin Rheumatol. 2006;25:639–44. 16365690 10.1007/s10067-005-0138-5

[14] Fautrel B Moël GL Saint-Marcoux B. Diagnostic value of ferritin and glycosylated ferritin in adult onset Still’s disease. 2001;9. 11246670

[15] Ohta A Yamaguchi M Tsunematsu T. Adult Still’s disease: a multicenter survey of Japanese patients. J Rheumatol. 1990;17:1058–63. 2213780

[16] Fautrel B. Adult-onset Still disease. Best Pract Res: Clin Rheumatol. 2008;22:773–92. 19028363 10.1016/j.berh.2008.08.006

[17] Zandman-Goddard G Shoenfeld Y. Ferritin in autoimmune diseases. Autoimmun Rev. 2007;6:457–63. 17643933 10.1016/j.autrev.2007.01.016

[18] Choi J-H Suh C-H Lee Y-M. Serum cytokine profiles in patients with adult onset Still’s disease. J Rheumatol. 2003;30:2422–7. 14677188

[19] Lee MH Means RT. Extremely elevated serum ferritin levels in a university hospital: associated diseases and clinical significance. Am J Med. 1995;98:566–71. 7778572 10.1016/s0002-9343(99)80015-1

[20] Efthimiou P. Diagnosis and management of adult onset Still’s disease. Ann Rheum Dis. 2006;65:564–72. 16219707 10.1136/ard.2005.042143 PMC1798146

[21] Cunha BA. Fever of unknown origin (FUO): diagnostic importance of serum ferritin levels. Scand J Infect Dis. 2007;39(6–7):651–2. 17577841 10.1080/00365540601169729

[22] Kim SE Kim UJ Jang MO. Diagnostic use of serum ferritin levels to differentiate infectious and noninfectious diseases in patients with fever of unknown origin. Dis Markers. 2013;34:211–8. 23324584 10.3233/DMA-130962 PMC3810234

[23] Moore C Ormseth M Fuchs H. Causes and significance of markedly elevated serum ferritin levels in an academic medical center. J Clin Rheumatol. 2013;19:324–8. 23965472 10.1097/RHU.0b013e31829ce01f

[24] Sackett K Cunderlik M Sahni N. Extreme hyperferritinemia: causes and impact on diagnostic reasoning. Am J Clin Pathol. 2016;145:646–50. 27247369 10.1093/ajcp/aqw053

[25] Pouchot J Sampalis JC Myhal D. Adult Still’s disease: manifestations, disease course, and outcome in 62 patients. Medicine. 1991;70:118–36. 2005777

[26] Ohta A Yamaguchi M Akizuri M. Adult Still’s disease: part I. Manifestations and complications in sixty-five cases in France. J Rheumatol. 1990;17:1058–63.2213780

